# Interaction between the non-alcoholic fatty liver disease fibrosis score and vitamin D deficiency on left ventricular hypertrophy and impaired diastolic function in patients with type 2 diabetes mellitus

**DOI:** 10.1186/s13098-025-01808-3

**Published:** 2025-08-01

**Authors:** Yun-Ming Li, Jia-Yi Huang, Ran Guo, Shi-Ming Li, Cong Chen, Min Wu, Run Wang, Ming-Ya Liu, Kai-Hang Yiu

**Affiliations:** 1 Health Science Center, Shenzhen University, Shenzhen, China; 2 Division of Cardiology, Department of Medicine, The University of Hong Kong Shen Zhen Hospital, Shenzhen, China; 3 Division of Cardiology, Department of Medicine, The University of Hong Kong, Queen Mary Hospital, Room 1929B/K1931, Block K, Hong Kong, China

**Keywords:** Non-alcoholic fatty liver disease fibrosis score, Vitamin D deficiency, Left ventricular hypertrophy, Left ventricular diastolic dysfunction, Type 2 diabetes mellitus

## Abstract

**Aims:**

The present study aimed to evaluate the effect of Non-Alcoholic Fatty Liver Disease Fibrosis Score (NFS), vitamin D deficiency, and their interaction on the Left Ventricle (LV) structure and diastolic function in type 2 diabetes mellitus (T2DM) patients.

**Methods:**

A total of 595 T2DM patients were recruited and stratified according to NFS grades (low, intermediate, and high) and the level of serum 25(OH)D (with and without vitamin D deficiency). Parameters of LV structure and diastolic dysfunction were measured by echocardiography. The association between NFS and LV structure and diastolic function was assessed using multivariable linear regression models stratified by vitamin D levels.

**Results:**

Left ventricular hypertrophy (LVH) was more prevalent in patients with high NFS compared to those with low and intermediate NFS (41.0 vs 14.0% and 9.0%, *P* < 0.001). The average E/e′ was higher in patients with intermediate and high NFS, as compared to those with low NFS. Within the high NFS group, patients with vitamin D deficiency exhibited significantly higher left ventricular mass index (LVMI) and average E/e′ compared to those without vitamin D deficiency. An interaction between vitamin D and NFS groups was found on both LVMI (*P* for interaction = 0.008) and average E/e′ (*P* for interaction = 0.001).

**Conclusions:**

NFS and vitamin D deficiency are associated with an increased risk of LVH and impaired diastolic function in patients with T2DM. Notably, the impact of vitamin D deficiency on these parameters is more pronounced in individuals with a high NFS score.

**Supplementary Information:**

The online version contains supplementary material available at 10.1186/s13098-025-01808-3.

## Introduction

Type 2 diabetes mellitus (T2DM) is associated with an increased risk of heart failure [1], with mounting evidence demonstrating adverse left ventricular (LV) remodeling in affected patients, including left ventricular hypertrophy (LVH) and LV diastolic dysfunction, irrespective of underlying coronary artery disease [2–4]. Despite widespread recognition of this phenomenon, the precise mechanisms underlying these alterations remain incompletely understood. Given the escalating burden of T2DM and its complications [5], identifying potential risk factors and their interrelationships is critical for effective risk stratification and treatment planning.

The co-occurrence of T2DM and metabolic dysfunction-associated steatotic liver disease (MASLD) is frequently observed and known to be a notable clinical association [6, 7]. Additionally, the presence of MASLD in T2DM patients is linked to unfavorable cardiovascular outcomes [8–10]. The NAFLD Fibrosis Score (NFS), a non-invasive marker, is widely used to predict fibrosis and fibrosis progression in patients with MASLD [11]. Some clinical studies suggested that greater NFS was associated with LV diastolic dysfunction and myocardial remodeling in patients with MASLD and T2DM [12–14]. Furthermore, the prevalence of vitamin D deficiency among patients with T2DM ranges from 60 to 90% based on available studies [15–17]. Despite conflicting opinions on the association between vitamin D deficiency and adverse cardiac remodeling as well as impaired diastolic function [18–21], the potential interplay between vitamin D deficiency and its interaction with NFS in the development of LVH and diastolic dysfunction remains unexplored. Our research aimed to conduct a cross-sectional analysis employing echocardiography to investigate the interactive effects of NFS and vitamin D levels on the prevalence of LVH and LV diastolic dysfunction in individuals diagnosed with T2DM.

## Methods

### Study population

A total of 626 patients with T2DM were consecutively recruited between January 2020 and May 2022 from the University of Hong Kong Shenzhen Hospital, Shenzhen, China. T2DM was diagnosed according to the World Health Organization criteria [22]. Patients with an alcohol intake >140 g/week for men and 70 g/week for women, and a history of heart failure or liver cirrhosis were excluded (n = 31). Finally, a total of 595 T2DM patients were included in the final analysis. The study was conducted according to the principles of the Declaration of Helsinki and was approved by the ethics committee of the University of Hong Kong Shenzhen Hospital, Shenzhen, China. Informed consent was obtained from all patients.

### Clinical data

Baseline clinical data were retrieved from the electronic medical records. Blood pressure was measured after resting for at least 5 min. Hypertension was defined as resting systolic or diastolic blood pressure above 140 or 90 mmHg respectively at two clinic visits or prescription of antihypertensive medication [23]. Body weight and height were measured, and Body mass index (BMI) was calculated as weight divided by height in meters squared (m^2^). Conventional cardiovascular risk factors such as a history of smoking, drinking, hypertension, and hypercholesteremia were documented. A medication history of statins, antihypertensive, and antidiabetic agents was retrieved. A fasting blood sample was obtained to measure glycated hemoglobin (HbA1c), total cholesterol (TC), total triglyceride (TG), high-density lipoprotein cholesterol (HDL-c), low-density lipoprotein cholesterol (LDL-c), plasma albumin (ALB), alanine aminotransferase (ALT), aspartate aminotransferase (AST), platelet (PLT) and 25(OH)D.

### Non-invasive markers of liver fibrosis and vitamin D deficiency

The NFS was calculated by: −1.675 + 0.037 *age (years) + 0.094 *BMI (kg/m^2^) + 1.13 * impaired fasting glucose/diabetes (yes = 1, no = 0) + 0.99 * AST/ALT ratio—0.013*platelet (* 10^9^ /L)—0.66 * albumin (g/dL). Two widely used cut-off points of NFS were selected to categorize the participants into three groups: those with low NFS (<−1.455), intermediate NFS (−1.455 ~ 0.676), and high NFS (>0.676) [24]. According to vitamin D requirements for optimal human health, vitamin D deficiency is defined as serum 25(OH)D concentrations lower than 50 nmol/l (20 ng/ml) respectively [25]. So, we defined serum 25(OH)D concentrations greater than 50 nmol/l (20 ng/ml) as without vitamin D deficiency in this study.

### Echocardiography

Transthoracic echocardiographic examination was performed in all patients using a Vivid E9, General Electric Vingmed Ultrasound machine (Milwaukee, WI, USA), with the patient lying in the lateral decubitus position. A 3.5-MHz transducer was used to obtain images that were digitally stored in cine-loop format with three cardiac cycles. The dimensions of the left ventricular (LV) chamber were measured according to the current recommendations. Interventricular septal dimension at end-diastole (IVSd), and LV posterior wall thickness at end-diastole (LVPWd) were measured using the two-dimensional echocardiography guided M-mode approach from the LV long axis view. Relative wall thickness (RWT) was calculated as two times the posterior wall thickness at end-diastole divided by left ventricular internal dimensions at end-diastole. Increased RWT was defined as RWT >0.42, and normal RWT as ≤0.42 [26]. Left ventricular mass (LVM) was calculated according to the Devereux formula [27], and left ventricular mass index (LVMI) was calculated as LVM divided by body surface area. Left ventricular hypertrophy (LVH) was defined as LVMI >115 g/m^2^ in men and LVMI >95 g/m^2^ in women. LV structural patterns were defined as normal (normal LVMI and normal RWT), concentric remodeling (normal LVMI and increased RWT), concentric hypertrophy (LVH and increased RWT), and eccentric hypertrophy (LVH and normal RWT).LV volume at the end of diastole (LVEDV), LV volume at the end of systole (LVESV) and left ventricular ejection fraction (LVEF) were determined from apical four and two-chamber views using a modified Simpson’s biplane method. Peak velocity in early (E-wave) and late (A-wave) diastole of mitral valve inflow was measured by pulsed-wave Doppler of the mitral valve inflow in apical four-chamber view and E/A ratio calculated. Pulsed wave tissue Doppler imaging was used to measure the early diastolic velocity of the mitral valve annulus with the sample volume placed at the septum (e′-sep) and lateral (e′-lat) annulus of the mitral valve, and Average E/e′ calculated. LV diastolic function was defined according to the ASE guideline (Average E/e′ > 14) [28].

### Statistical analysis

Continuous variables with a normal distribution are presented as mean ± standard deviation (SD), and skewed variables were expressed as median (interquartile range [IQR]). One-way ANOVA and non-parametric tests were used appropriately to examine the difference among groups of NFS and vitamin D. Categorical variables are presented as frequencies and percentages and were compared using the χ^2^ test. Univariable linear regression analyses were used to assess the relationships of NFS with laboratory and echocardiography data. Multivariable linear regression models with an interaction term were performed to test the influence of the level of vitamin D on the association between echocardiography data and NFS. All statistical analyses were performed using the statistical package SPSS for Windows (Version 23.0). The diagram software used for this article is Graphpad Prism9 and SPSS (version 23.0). A two-sided *P* value <0.05 was considered statistically significant.

## Results

### Clinical characteristics of the group of NFS

Our study included a total of 595 patients, of whom 39.5% were women. Baseline characteristics of the study population according to NFS levels are presented in Table 1. Patients in the intermediate and high NFS groups were found to be older, had a higher prevalence of hypertension, longer duration of diabetes, and had higher DBP, HR, HbA1c, TC, LDL-C, ALT, ALB, and platelet numbers when compared with those in the low NFS group (all *P* < 0.001). Additionally, 25(OH)D levels in the intermediate and high NFS groups were significantly increased compared with those in the low NFS group (*P* < 0.001).

**Table 1:** Baseline characteristics of the study population according to low, intermediate, and high Non-Alcoholic Fatty Liver Disease Fibrosis Score

Variables	Low NFS	Intermediate NFS	High NFS	*P*
N (%)	185 (31.1)	337 (56.6)	73 (12.3)	595
Men (%)	114 (61.6)	212 (62.9)	34 (46.6)	0.033
Age (years)	47.14 ± 11.99	60.77 ± 12.18	72.21 ± 12.70	<0.001
BMI (kg/m^2^)	24.48 ± 3.75	24.71 ± 3.96	25.48 ± 4.25	0.182
SBP (mmHg)	130.49 ± 19.16	131.5 ± 19.43	135.88 ± 23.07	0.139
DBP (mmHg)	80.13 ± 11.21	76.18 ± 10.31^a^	74.63 ± 10.43^b^	<0.001
HR (bpm)	88.46 ± 12.52	82.33 ± 13.76	77.14 ± 13.02	<0.001
Medical history				
Diabetes duration (years)	4 (0.63–10)	9 (4–13)	10 (4.25–20)	<0.001
Hypertension, n (%)	70 (37.8)	157 (46.6)	50 (68.5)	<0.001
Hypercholesterolaemia, n (%)	74 (40)	123 (36.5)	21 (28.8)	0.240
Drinker, n (%)	23 (12.4)	40 (11.9)	7 (9.6)	0.812
Smoker, n (%)	53 (28.6)	83 (24.6)	11 (15.1)	0.075
Medications				
ACEI/ARB, n (%)	37 (20.1)	81 (24)	30 (41.1)	0.002
b-Blocker, n (%)	14 (7.6)	58 (17.2)	23 (31.5)	<0.001
CCB, n (%)	37 (20.1)	84 (24.9)	26 (36.1)	0.029
Metformin, n (%)	94 (50.8)	200 (59.3)	33 (45.2)	0.035
SGLT2i, n (%)	28 (15.2)	67 (19.9)	20 (27.8)	0.069
Statin, n (%)	30 (16.2)	84 (24.9)	37 (51.4)	<0.001
Blood chemistry				
HbA1C (%)	10.08 ± 2.37	9.53 ± 2.29	8.12 ± 1.94	<0.001
TG (mmol/L)	1.97 (1.23–3.5)	1.89 (1.26–2.99)	1.74 (1.16–2.96)	0.303
TC (mmol/L)	5.25 ± 1.78	4.69 ± 1.75^a^	4.16 ± 1.42^b^	<0.001
LDL-c (mmol/L)	3.06 ± 1.11	2.75 ± 1.04	2.31 ± 1.02	<0.001
HDL-c (mmol/L)	1.02 ± 0.35	1.05 ± 0.29	1.12 ± 0.37	0.116
ALT (U/L)	22.1 (14.75–35.9)	17.4 (12.45–26)	12 (9.25–20.9)	<0.001
AST (U/L)	16.9 (13.85–23.6)	17.8 (14.3–22.9)	18.3 (14.55–22.7)	0.637
ALB (g/L)	43.46 ± 4.46	41.48 ± 3.57	39.83 ± 4.09	<0.001
PLT (10^9/L)	287.97 ± 67.40	213.8 ± 45.68	154.21 ± 51.96	<0.001
25(OH)D(ng/ml)	20.85 ± 8.45	24.44 ± 9.40^a^	24.03 ± 10.99^b^	<0.001
IVSd (mm)	9.38 ± 1.37	9.55 ± 1.51	9.84 ± 1.72	0.003
LVPWd (mm)	9.39 ± 1.14	9.47 ± 1.36	10.14 ± 2.89	0.114
RWT	0.42 (0.38–0.45)	0.41 (0.38–0.46)	0.42 (0.38–0.46)	0.740
LVMI (g/m^2^)	81.04 ± 16.79	85.79 ± 20.11	96.48 ± 23.81	<0.001
LVEDV (ml)	90.82 ± 21.71	96.5 ± 22.04	103.12 ± 22.52	0.001
LVESV (ml)	32.03 ± 10.43	33.51 ± 11.05	36.86 ± 11.18	0.020
LVEF (%)	65.43 ± 5.26	65.61 ± 6.51	65.29 ± 6.61	0.907
E (cm/s)	68.81 ± 17.15	66.51 ± 19.07	71.51 ± 22.41	0.112
A (cm/s)	75.66 ± 26.30	84.08 ± 22.73	95.09 ± 25.00	<0.001
E/A	1.0 ± 0.38	0.81 ± 0.27	0.73 ± 0.22	<0.001
e′-sep (cm/s)	7.55 ± 2.49	6.46 ± 2.37	5.53 ± 2.06	<0.001
e′-lat (cm/s)	10.89 ± 2.91	9.08 ± 3.05	8.00 ± 2.78	<0.001
Average E/e′	9.92 ± 4.26	11.46 ± 5.50	14.03 ± 6.15	<0.001

A, trans-mitral late diastolic peak velocity; ACEI, angiotensin-converting-enzyme inhibitor; ALB, plasma albumin; ALT, alanine aminotransferase; AST, aspartate aminotransferase; ARB, angiotensin receptor blocker; BMI, body mass index; CCB, calcium channel blockers; DBP, diastolic blood pressure; E, trans-mitral early diastolic peak velocity; e′, early diastolic peak velocity of mitral valve at septal or lateral annulus; HbA1c, haemoglobin A1 c; HDL-C, high-density lipoprotein cholesterol; HR, heart rate; IVSd, inter-ventricular septal dimension at end-diastole; LDL-C, low-density lipoprotein cholesterol; LVEDV, LV end-diastolic volume; LVEF, LV ejection fraction; LVESV, LV end-systolic volume; LVMI, LV mass index; LVPWd, LV posterior wall thickness at end-diastole; PLT, platelet; RWT, relative wall thickness; SBP, systolic blood pressure; SGLT2i, Sodium-glucose cotransporter 2 inhibitors; TC, total cholesterol; TG, total triglyceride; 25(OH)D, serum 25-hydroxyvitamin

^*^ Note: For continuous variables, mean ± standard deviation (One-way ANOVA) or median [interquartile range] (Mann–Whitney U test) were used; Categorical variables were measured using n (%) (chi-square test)

^a^*P* < 0.05 between the low NFS group and the intermediate NFS group

^b^*P* < 0.05 between the low NFS group and the high NFS group

### Association of the NFS with LV structure and diastolic function

**Fig. 1:**
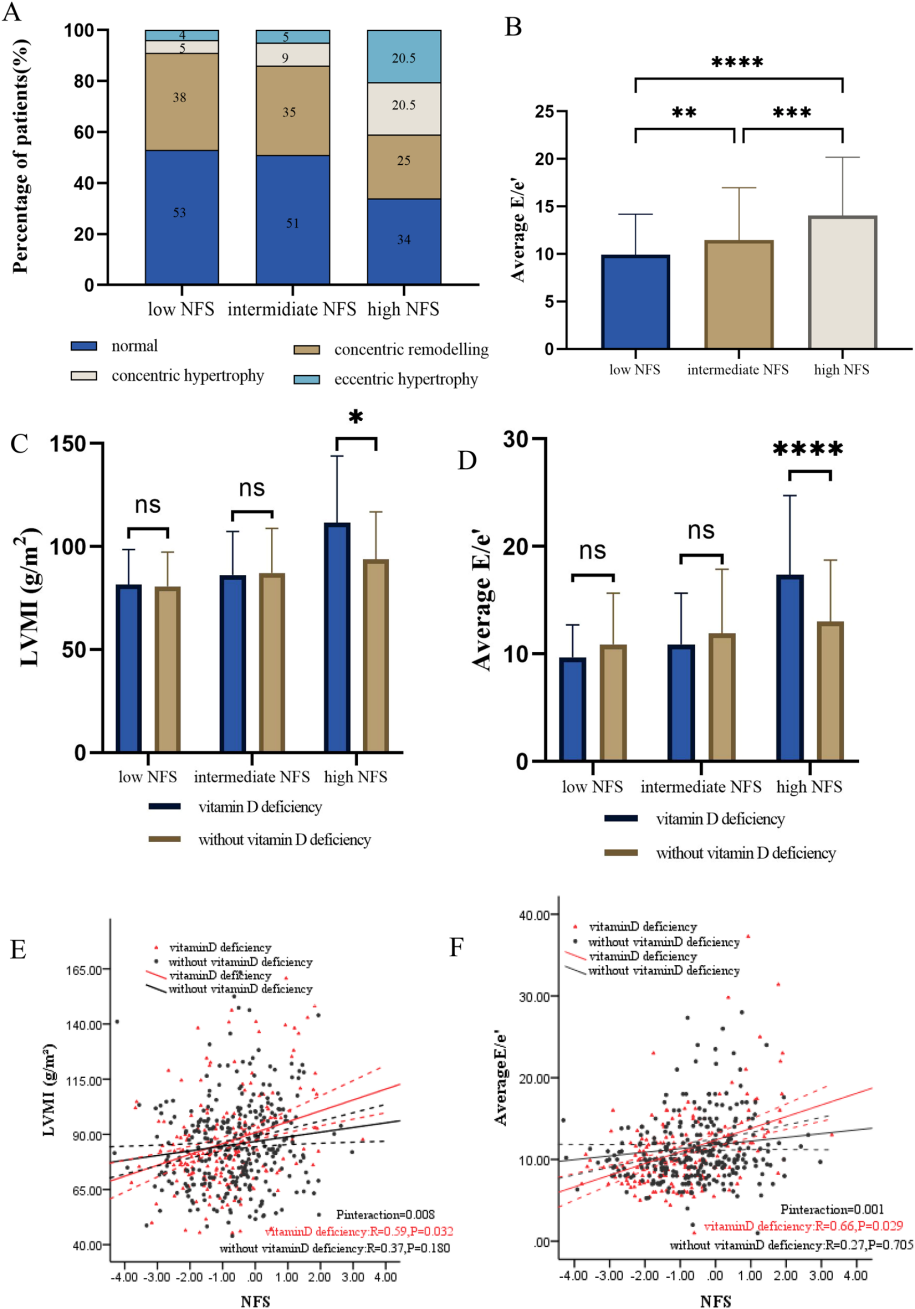
**A** Distribution of various patterns of left ventricular hypertrophy in the NFS group. **B** Distribution of the NFS group with Average E/e′. (C&D) Association of the NFS with LVMI and Average E/e′ in the group of vitamin D and NFS. (E&F) An interaction effect of vitamin D group on the association between the NFS and LVMI, Average E/e′. Abbreviations: Average E/e′, E-wave transmitral velocity to early diastolic velocity at tissue-Doppler imaging; LVMI, left ventricular mass index; NFS, the NAFLD Fibrosis Score

Parameters of LV structure and diastolic dysfunction were summarized in Table 1. LV structure parameters including IVSd, LVMI, LVEDV, and LVESV in the groups of intermediate and high NFS were significantly higher than in the low NFS group. Nonetheless, LVH (defined as eccentric hypertrophy and concentric hypertrophy) was more common in the high NFS patients than in the low and intermediate NFS patients (41.0 vs 14.0% and 9.0%, *P* < 0.001; Fig. 1A). Patients stratified into intermediate and high NFS exhibited compromised LV diastolic function parameters, characterized by increased Average E/e′ and decreased E/A, e′-sep, and e′-lat values, in comparison to patients with low NFS. These findings distinctly indicate the presence of diastolic dysfunction in the former group (Fig. 1B).

### Clinical characteristics and Echocardiographic parameters of vitamin D group

The baseline characteristics of the study population according to the vitamin D group are shown in Table 2. Patients in the vitamin D deficiency group were younger, and had higher BMI, DBP, HR, HbA1c, TG, TC, PLT, and shorter duration of diabetes than those without vitamin D deficiency. Patients without vitamin D deficiency had a higher HDL level, while LDL-C, ALT, AST, and ALB were similar to those in the vitamin D deficiency group. Patients with vitamin D deficiency had higher LVPWd, LVESV, and e′-lat compared to those without vitamin D deficiency. Nonetheless, the prevalence of LVH and average E/e` ratio were similar between the two groups.

**Table 2:** Baseline characteristics of the study population according to the group of vitamin D

Variables	Vitamin D deficiency	Without vitamin D deficiency	*P*
N (%)	236 (40.0)	359 (60.0)	595
Men (%)	145 (61.4)	215 (59.9)	0.705
Age (years)	53.49 ± 15.23	61.16 ± 13.14	<0.001
BMI (kg/m^2^)	25.64 ± 4.26	24.14 ± 3.60	<0.001
SBP (mmHg)	132.34 ± 19.16	131.32 ± 20.32	0.539
DBP (mmHg)	79.00 ± 10.89	76.06 ± 10.58	0.001
HR (bpm)	87.14 ± 13.49	81.27 ± 13.47	<0.001
*Medical history*			
Diabetes duration (years)	5 (1–10)	9 (3–14)	<0.001
Hypertension, n (%)	106 (44.9)	171 (47.6)	0.516
Hyperlipidemia, n (%)	93 (39.4)	125 (34.8)	0.256
Drinker, n (%)	27 (11.4)	43 (12.0)	0.060
Smoker, n (%)	68 (28.8)	79 (22.9)	0.842
Medications			
ACEI/ARB, n (%)	55 (23.4)	93 (25.9)	0.491
b-Blocker, n (%)	27 (11.4)	68 (18.9)	0.015
CCB, n (%)	63 (26.9)	84 (23.4)	0.331
Metformin, n (%)	123 (52.1)	204 (56.8)	0.259
SGLT2i, n (%)	36 (15.3)	79 (22.1)	0.042
Statin, n (%)	41 (17.4)	110 (30.6)	<0.001
*Blood chemistry*			
HbA1C (%)	9.94 ± 2.53	9.25 ± 2.18	0.001
TG (mmol/L)	2.20(1.33–3.36)	1.74(1.20–2.82)	<0.001
TC (mmol/L)	5.13 ± 2.28	4.59 ± 1.26	0.001
HDL-c (mmol/L)	0.99 ± 0.31	1.09 ± 0.32	<0.001
LDL-c (mmol/L)	2.81 ± 1.11	2.78 ± 1.07	0.787
ALT (U/L)	19.4 (11.93–28.90)	17.3 (12.6–27)	0.510
AST (U/L)	17.5 (13.9–22.1)	17.9 (14.3–23.3)	0.315
ALB (g/L)	41.5 ± 4.77	42.15 ± 3.58	0.078
PLT (10^9/L)	240.32 ± 74.07	222.46 ± 65.30	0.002
NFS	−1.01 ± 1.36	−0.64 ± 1.22	0.001
*Echocardiography*			
IVSd (mm)	9.61 ± 1.60	9.5 ± 1.43	0.395
LVPWd (mm)	9.74 ± 1.99	9.4 ± 1.3	0.019
RWT	0.42(0.38–0.47)	0.42(0.38–0.46)	0.387
LVMI (g/m^2^)	86.40 ± 21.36	85.46 ± 19.60	0.606
LVEDV (ml)	97.47 ± 23.52	94.6 ± 21.43	0.177
LVESV (ml)	34.83 ± 11.07	32.68 ± 10.84	0.040
LVEF (%)	64.88 ± 5.62	65.92 ± 6.48	0.059
E (cm/s)	69.64 ± 21.98	66.7 ± 16.89	0.106
A (cm/s)	82.02 ± 27.62	83.57 ± 22.86	0.493
E/A	0.89 ± 0.33	0.84 ± 0.30	0.051
e′-sep (cm/s)	6.91 ± 2.77	6.49 ± 2.22	0.066
e′-lat (cm/s)	9.88 ± 3.26	9.19 ± 3.02	0.023
Average E/e′	11.07 ± 4.93	11.52 ± 5.67	0.365

A, trans-mitral late diastolic peak velocity; ACEI, angiotensin-converting-enzyme inhibitor; ALB, plasma albumin; ALT, alanine aminotransferase; AST, aspartate aminotransferase; ARB, angiotensin receptor blocker; BMI, body mass index; CCB, calcium channel blockers; DBP, diastolic blood pressure; E, trans-mitral early diastolic peak velocity; e′, early diastolic peak velocity of mitral valve at septal or lateral annulus; HbA1c, haemoglobin A1 c; HDL-C, high-density lipoprotein cholesterol; HR, heart rate; IVSd, inter-ventricular septal dimension at end-diastole; LDL-C, low-density lipoprotein cholesterol; LVEDV, LV end-diastolic volume; LVEF, LV ejection fraction; LVESV, LV end-systolic volume; LVMI, LV mass index; LVPWd, LV posterior wall thickness at end-diastole; PLT, platelet; RWT, relative wall thickness; SBP, systolic blood pressure; SGLT2i, Sodium-glucose cotransporter 2 inhibitors; TC, total cholesterol; TG, total triglyceride

^*^ Note: For continuous variables, mean ± standard deviation (One-way ANOVA) or median [interquartile range] (Mann–Whitney U test) were used; Categorical variables were measured using n (%) (chi-square test)

### Clinical characteristics and Echocardiographic parameters about the group of vitamin D and NFS

The clinical features of the patients included in our study were systematically characterized based on different combinations of vitamin D levels and NFS, as outlined in Table 3. Notably, no substantial differences were detected in LVMI or Average E/e′ between patients with or without vitamin D deficiency in the intermediate and low NFS groups. However, within the high NFS group, patients with vitamin D deficiency displayed significantly higher LVMI and Average E/e′ values compared to those without vitamin D deficiency, as highlighted in Fig. 1C, D, respectively.

**Table 3:** Cardiometabolic and cardiac structure–function data stratified by the group of NFS and vitamin D

Variable	Low NFS		*p*	Intermediate NFS		*p*	High NFS		*p*
	Vitamin D deficiency	Without vitamin D deficiency		Vitamin D deficiency	Without vitamin D deficiency		Vitamin D deficiency	Without vitamin D deficiency	
N (%)	94 (50.0)	94 (50.0)		116 (34.4)	221 (65.6)		28 (38.4)	45 (61.6)	
Men (%)	59 (64.1)	55 (59.1)	0.485	75 (64.7)	137 (62.0)	0.630	11 (39.3)	23 (51.1)	0.325
Age (years)	44.52 ± 12.38	50.91 ± 10.75	<0.001	56.78 ± 13.14	62.86 ± 11.11	<0.001	69.29 ± 13.73	74.02 ± 11.81	0.122
BMI (kg/m^2^)	25.19 ± 4.13	23.77 ± 3.21	0.010	25.83 ± 4.18	24.13 ± 3.72	<0.001	26.34 ± 4.98	24.95 ± 3.68	0.175
SBP (mmHg)	132.98 ± 17.98	128.02 ± 20.06	0.079	129.42 ± 17.80	132.57 ± 20.18	0.160	142.14 ± 24.85	131.98 ± 21.24	0.067
DBP (mmHg)	81.73 ± 11.62	78.55 ± 10.62	0.053	77.55 ± 9.13	75.46 ± 10.83	0.079	75.93 ± 13.29	73.82 ± 8.24	0.405
HR (bpm)	91.22 ± 11.07	85.76 ± 13.31	0.003	86.3 ± 13.9	80.23 ± 13.24	<0.001	77.32 ± 13.70	77.02 ± 12.73	0.925
*Medical history*									
Diabetes duration (years)	2 (0.5–6)	6 (1–10)	0.010	6 (2–11)	10 (4–15)	0.014	16 (5.25–20)	10 (3.25–20)	0.219
Hypertension, n (%)	35 (38.0)	35 (37.6)	0.954	50 (43.1)	107 (48.4)	0.353	21 (75)	29 (64.4)	0.345
Hyperlipidemia, n (%)	39 (42.4)	35 (37.6)	0.509	46 (39.7)	77 (34.8)	0.383	8 (28.6)	13 (28.9)	0.977
Drinker, n (%)	9 (9.8)	14 (15.1)	0.277	16 (13.8)	24 (10.9)	0.429	2 (7.1)	5 (11.1)	0.576
Smoker, n (%)	28 (30.4)	25 (26.9)	0.593	34 (29.3)	49 (22.2)	0.148	6 (21.4)	5 (11.1)	0.231
*Medications*									
ACEI/ARB, n (%)	19 (20.9)	18 (19.4)	0.796	24 (20.7)	57 (25.8)	0.298	12 (42.9)	18 (40)	0.809
b-Blocker, n (%)	8 (8.7)	6 (6.5)	0.564	12 (10.3)	46 (20.8)	0.016	7 (25)	16 (35.6)	0.345
CCB, n (%)	24 (26.4)	13 (14.0)	0.036	30 (25.9)	54 (24.4)	0.773	9 (33.3)	17 (37.8)	0.704
Metformin, n (%)	40 (43.5)	54 (58.1)	0.047	65 (56)	135 (61.1)	0.370	18 (64.3)	15 (33.3)	0.010
SGLT2i, n (%)	10 (10.9)	18 (19.6)	0.101	19 (16.4)	48 (21.7)	0.243	7 (25.9)	13 (28.9)	0.786
Statin, n (%)	10 (10.9)	20 (21.5)	0.050	19 (16.4)	65 (29.4)	0.009	12 (44.4)	25 (55.6)	0.361
*Blood chemistry*									
HbA1C (%)	10.29 ± 2.46	9.88 ± 2.27	0.267	10.09 ± 2.55	9.2 ± 2.10	0.002	8.21 ± 2.14	8.06 ± 1.81	0.756
TG (mmol/L)	2.23 (1.30–4.95)	1.64 (1.20–3.17)	0.055	2.27 (1.46–3.31)	1.75 (1.21–2.64)	0.001	2.14 (1.17–2.67)	1.59 (1.14–3.29)	0.591
TC (mmol/L)	5.38 ± 2.11	5.13 ± 1.38	0.344	5.10 ± 2.53	4.49 ± 1.19	0.015	4.47 ± 1.80	3.96 ± 1.10	0.145
LDL-c (mmol/L)	2.93 ± 1.02	3.2 ± 1.19	0.112	2.75 ± 1.15	2.72 ± 0.99	0.608	2.49 ± 1.18	2.19 ± 0.91	0.228
HDL-c (mmol/L)	0.96 ± 0.35	1.09 ± 0.34	0.014	0.98 ± 0.26	1.08 ± 0.30	0.003	1.10 ± 0.38	1.13 ± 0.36	0.771
ALT (U/L)	22.45 (15.08–35.95)	20.8 (14.1–35.85)	0.520	18.25 (11.98–27.4)	17.3 (12.55–25.7)	0.607	10.85 (8.08–17.43)	15.2 (10.65–21.7)	0.036
AST (U/L)	17.05 (13.93–22.25)	16.9 (13.5–23.95)	0.890	17.6 (14–22.53)	18 (14.5–23.1)	0.677	17.4 (12.58–21.88)	18.9 (15.05–25.85)	0.098
ALB (g/L)	43.1 ± 4.99	43.81 ± 3.86	0.278	40.8 ± 4.03	41.73 ± 3.29	0.020	38.96 ± 5.22	40.37 ± 3.15	0.153
PLT (10^9/L)	290.34 ± 73.7	285.62 ± 60.85	0.636	219.86 ± 44.52	211.06 ± 45.16	0.097	162.29 ± 63.45	149.18 ± 43.37	0.298
*Echocardiography*									
IVSd (mm)	9.53 ± 1.3	9.25 ± 1.42	0.218	9.42 ± 1.67	9.62 ± 1.42	0.285	10.56 ± 1.81	9.38 ± 1.51	0.005
LVPWd (mm)	9.58 ± 1.05	9.22 ± 1.21	0.048	9.48 ± 1.38	9.46 ± 1.34	0.889	11.15 ± 4.20	9.5 ± 1.25	0.020
RWT	0.42(0.40–0.48)	0.41(0.38–0.44)	0.025	0.40 (0.36–0.46)	0.42 (0.38–0.46)	0.137	0.43(0.40–0.48)	0.40(0.36–0.44)	0.057
LVMI (g/m^2^)	81.65 ± 17.09	80.50 ± 16.61	0.678	84.73 ± 19.97	86.34 ± 20.21	0.513	105.94 ± 26.67	90.62 ± 20.02	0.015
LVEDV (ml)	92.11 ± 20.08	89.62 ± 23.20	0.511	99.18 ± 23.43	95.12 ± 21.23	0.151	105.57 ± 29.78	101.56 ± 16.63	0.509
LVESV (ml)	32.81 ± 8.53	31.29 ± 11.97	0.405	35.3 ± 11.19	32.6 ± 10.90	0.056	38.73 ± 15.56	35.72 ± 7.39	0.403
LVEF (%)	64.68 ± 4.49	66.13 ± 5.82	0.092	64.84 ± 5.54	66.01 ± 6.93	0.141	65.52 ± 8.27	65.14 ± 5.38	0.820
E(cm/s)	70.1 ± 19.14	67.63 ± 15.13	0.379	67.57 ± 22.87	65.97 ± 16.83	0.534	76.26 ± 24.89	68.39 ± 20.32	0.158
A (cm/s)	78.39 ± 31.93	73.16 ± 19.74	0.224	79.77 ± 23.93	86.28 ± 21.84	0.020	101.48 ± 19.98	91 ± 27.20	0.102
E/A	1.01 ± 0.35	0.99 ± 0.40	0.786	0.85 ± 0.30	0.80 ± 0.25	0.117	0.73 ± 0.23	0.73 ± 0.22	0.992
e′-sep (mm/s)	7.77 ± 2.71	7.35 ± 2.27	0.302	6.86 ± 2.78	6.26 ± 2.11	0.055	4.83 ± 1.51	5.98 ± 2.25	0.023
e′-lat (mm/s)	11.27 ± 3.00	10.55 ± 2.81	0.166	9.56 ± 3.21	8.83 ± 2.94	0.071	7.41 ± 2.26	8.36 ± 3.03	0.208
Average E/e′	9.70 ± 3.05	10.14 ± 5.16	0.535	10.62 ± 4.48	11.87 ± 5.92	0.067	16.69 ± 6.93	12.36 ± 5.02	0.005

A, trans-mitral late diastolic peak velocity; ACEI, angiotensin-converting-enzyme inhibitor; ALB, plasma albumin; ALT, alanine aminotransferase; AST, aspartate aminotransferase; ARB, angiotensin receptor blocker; BMI, body mass index; CCB, calcium channel blockers; DBP, diastolic blood pressure; E, trans-mitral early diastolic peak velocity; e′, early diastolic peak velocity of mitral valve at septal or lateral annulus; HbA1c, haemoglobin A1 c; HDL-C, high-density lipoprotein cholesterol; HR, heart rate; IVSd, inter-ventricular septal dimension at end-diastole; LDL-C, low-density lipoprotein cholesterol, LVEDV, LV end-diastolic volume; LVEF, LV ejection fraction; LVESV, LV end-systolic volume; LVMI, LV mass index; LVPWd, LV posterior wall thickness at end-diastole; PLT, platelet; RWT, relative wall thickness; SBP, systolic blood pressure; SGLT2i, Sodium-glucose cotransporter 2 inhibitors; TC, total cholesterol; TG, total triglyceride

^*^ Note: For continuous variables, mean ± standard deviation (One-way ANOVA) or median [interquartile range] (Mann–Whitney U test) were used; Categorical variables were measured using n (%) (chi-square test)

### Interaction of vitamin D deficiency on the relationship between NFS group and LVH, LV diastolic dysfunction

To determine whether an interaction exists between vitamin D deficiency and NFS with LVH and LV diastolic dysfunction in patients with T2DM, multivariable linear regression was conducted, as presented in Table 4. After adjusting for several covariates, including age, sex, BMI, SBP, HR, diabetes duration, HbA1C, TG, LDL-c, HDL-c, we observed a significant association between LVMI and increasing NFS in patients with vitamin D deficiency (*p* = 0.032), but not in those without vitamin D deficiency. We found a significant interaction between vitamin D and the NFS group on LVMI (*P* for interaction = 0.008, Fig. 1E). Additionally, the correlation between Average E/e′ and NFS was significantly stronger in patients with vitamin D deficiency than in those without vitamin D deficiency (*p* = 0.029). A significant interaction between vitamin D and the NFS group on Average E/e′ was also observed (*P* for interaction = 0.001, Fig. 1F).

**Table 4:** Associations with NFS by vitamin D group

Predictor	Without vitamin D deficiency		Vitamin D deficiency		Pinteraction
	β (95%CI)	*P*	β (95%CI)	*P*	
LVMI (g/m^2^)	0.10 (−0.77–4.06)	0.18	0.19 (0.26–5.74)	0.032	0.008
Average E/e′	0.03 (−0.62–0.92)	0.705	0.18 (0.07–1.28)	0.029	0.001

Average E/e′, E-wave transmitral velocity to early diastolic velocity at tissue-Doppler imaging; CI, confidence interval; LVMI, LV mass index;

The multivariable linear regression is adjusted for age, sex, body mass index, systolic blood pressure, heart rate, diabetes duration, haemoglobin-A1 c, total triglyceride, low-density lipoprotein cholesterol and high-density lipoprotein cholesterol.Further details are provided in Supplementary Tables S1 and S2

## Discussion

Our investigation revealed that the level of NFS was a significant independent predictor of both LVH and LV diastolic dysfunction in patients with T2DM. While vitamin D deficiency alone did not demonstrate a significant association with LVH or diastolic dysfunction, we observed a significant interaction between vitamin D deficiency and greater NFS, which was linked to poorer diastolic function and a higher prevalence of LVH when both factors were present.

Not only widely used as a marker of advanced fibrosis [11], NFS can reflect the risk of coronary atherosclerosis, cardiovascular events, and mortality in patients with MASLD [29–31]. Notably, NFS has also been linked to CVD risk factors in diverse populations, independent of MASLD status [32, 33]. In a prior study, higher NFS levels was associated with a higher prevalence of LVH and impaired LV diastolic function as measured by the E/A ratio and deceleration time (DT) in patients with T2DM [12]. Unlike these conventional parameters, our study utilized the average E/e′ ratio as the primary metric for assessing LV diastolic function. Additionally, a distinct study identified a link between the NFS and subclinical myocardial remodeling in patients diagnosed with T2DM, regardless of the presence of MASLD [34]. Our results are consistent with previous investigations that have identified NFS as a valuable predictor of LVH and LV diastolic performance in T2DM patients. The influence of the NFS on cardiac remodeling and diastolic dysfunction is likely mediated by various mechanisms, such as inflammation, oxidative stress, metabolic syndrome, immune modulation, and potentially other factors. However, the precise underlying mechanisms necessitate additional research to be comprehensively elucidated.

The prevalence of vitamin D deficiency is progressively increasing and numerous clinical investigations have consistently associated vitamin D deficiency with an elevated risk of cardiovascular disease [35, 36]. Low concentrations of vitamin D stores were thought to increase CVD risk not only by exacerbating subclinical inflammation, endothelial dysfunction, arterial stiffness, and hypercoagulability, but also by increasing the risk of established CVD risk factors (such as hypertension, diabetes, and dyslipidemia) [37]. However, the assertion that vitamin D deficiency may contribute to detrimental cardiac remodeling and diastolic function impairment remains a subject of controversy. In a study comprising 136 patients recently diagnosed with T2DM, it reduced vitamin D levels was independently correlated with LVMI [19]. Within an investigation comprising 280 adult patients with early-onset T2DM, lower levels of 25(OH)D was associated with LV diastolic dysfunction [20]. However, in a prospective investigation encompassing 711 community-dwelling volunteers, it was discerned that 25(OH)D exhibited a positive correlation with LV thickness and LVMI [18]. Furthermore, an additional study, which encompassed 95 patients diagnosed with T2DM, noted that individuals with vitamin D deficiency displayed similar left ventricular (LV) geometry, diastolic function, and LVEF when compared to those without vitamin D deficiency [21]. In the current study, patients with T2DM and concomitant vitamin D deficiency demonstrated no discernible differences in LV structure and LV diastolic function when contrasted with individuals without vitamin D deficiency.

Although NFS is linked with LVH and LV diastolic dysfunction, its detrimental impact is notably more pronounced in individuals with coexisting vitamin D deficiency. This observation implies the possibility of an incremental harmful mechanism responsible for cardiac remodeling in patients diagnosed with T2DM. There are several potential explanations for this phenomenon. Firstly, vitamin D is a potential negative modulator of proinflammatory cytokines release [38, 39]. Moreover, vitamin D deficiency can promote the occurrence of MASLD by promoting oxidative stress and systemic inflammatory response [40–42]. Consequently, inflammation and oxidative stress may detrimentally affect the function of cardiac muscles and blood vessels, resulting in cardiac remodeling and impaired diastolic function. Secondly, vitamin D plays a role in regulating various hormones, including calcium, phosphorus, and the renin–angiotensin–aldosterone system, which are essential for cardiovascular system regulation [43–45]. Deficiencies in vitamin D, combined with high NFS levels, may disrupt the normal functioning of these hormones. Thirdly, the NFS is composed of metabolic components whose effects go beyond the liver. The components of the NFS reflect downstream complications of systemic insulin resistance, obesity, and metabolic syndrome, each of which have been separately linked to increased cardiovascular risk. Additionally, vitamin D deficiency may also be associated with metabolic syndrome, especially insulin resistance and obesity [46–48]. While biological plausibility exists for both vitamin D deficiency and elevated NFS to independently influence cardiac remodeling, their relative contributions and potential interactions remain poorly understood due to current gaps in mechanistic studies. The specific mechanisms by which high NFS and vitamin D deficiency may promote LVH and impaired LV diastolic function in patients with T2DM require further investigation.

### Clinical implication

Our findings not only shed light to the underlying mechanism in the development of LVH and diastolic dysfunction T2DM patients, but potential clinical implication can also be derived from the present data. The use of NFS and Vitamin D level assessment is readily available with routine blood biochemistry and that the use of such can identify those who are at high risk of adverse LV remodeling and diastolic dysfunction. Studies have identified ways to improve NFS, such as weight reduction and statin therapy [49, 50] and should therefore be encouraged in patients with T2DM to potential improve their LV structure and diastolic dysfunction. In the same context, vitamin D deficiency can be easily reverse by adequate supplement and should thus be considered in patients with T2DM, especially those with concomitant high NFS. Future randomized study should be performed to evaluate the potential clinical benefit in managing NFS and vitamin D deficiency in patients with T2DM.

## Limitation

There are several limitations in our study. Firstly, an intrinsic deficiency of a cross-sectional study is that a cause-effect inference cannot be made. Secondly, LV diastolic function may not be accurately evaluated through only one parameter, Average E/e′. Finally, recent evidence indicates the incremental diagnostic value of speckle tracking echocardiography (STE) over conventional transthoracic echocardiography for detecting subclinical myocardial dysfunction in MASLD patients [51–53]. However, our study did not assess the effects of NFS and vitamin D deficiency on myocardial strain parameters assessed by STE in T2DM patients. Further studies are warranted to evaluate the impact of NFS and vitamin D status on STE-derived myocardial strain parameters in this population.

## Conclusion

The present study provides evidence supporting the link between NFS and vitamin D deficiency with the risk of LVH and impaired diastolic function in patients with T2DM. Notably, we observed a significant interaction between vitamin D deficiency and NFS groups, with a greater impact on LVMI and average E/e′ in individuals with a high NFS score. Our findings underscore the need for clinicians to evaluate both NFS and vitamin D levels in T2DM patients to better assess their cardiovascular health.

## Supplementary Information

Additional file 1.

## Data Availability

The datasets used and/or analysed during the current study are available from the corresponding author on reasonable request.
