# Supplementary material for: Interaction between the non-alcoholic fatty liver disease fibrosis score and vitamin D deficiency on left ventricular hypertrophy and impaired diastolic function in patients with type 2 diabetes mellitus
Source: Diabetol Metab Syndr. 2025 Aug 1;17:307. doi: 10.1186/s13098-025-01808-3 (PMC12315323; doi:10.1186/s13098-025-01808-3)
Supplement: Supplementary file 1 — Additional file 1. [file 13098_2025_1808_MOESM1_ESM.docx]

**Supplementary Table S1** Multivariable Linear Regression Analysis of LVMI by Vitamin D Status.

|  | vitamin D deficiency | | | without vitamin D deficiency | | |
| --- | --- | --- | --- | --- | --- | --- |
| Predictor | β | 95%CI | P | β | 95%CI | P |
| NFS | 0.19 | [0.26,5.74] | 0.032 | 0.10 | [-0.77,4.06] | 0.180 |
| Sex | 0.11 | [-1.19,11.56] | 0.110 | 0.07 | [-2.06,7.85] | 0.251 |
| Age(years) | 0.12 | [-0.10,0.46] | 0.216 | -0.02 | [-0.29,0.23] | 0.819 |
| SBP(mmHg) | 0.31 | [0.20,0.52] | ＜0.001 | 0.26 | [0.14,0.37] | ＜0.001 |
| BMI(kg/m2) | -0.08 | [-1.14,0.37] | 0.311 | -0.03 | [-0.86,0.5] | 0.603 |
| Diabetes duration (years) | 0.15 | [-0.01,0.84] | 0.053 | 0.08 | [-0.11,0.55] | 0.193 |
| HbA1C (%) | -0.10 | [-2.10,0.43] | 0.194 | -0.02 | [-1.26,0.95] | 0.782 |
| LDL-c (mmol/L) | -0.10 | [-4.95,0.83] | 0.162 | -0.03 | [-2.95,1.86] | 0.653 |
| HDL-c(mmol/L) | -0.19 | [-25.40,-2.60] | 0.016 | -0.01 | [-8.84,7.63] | 0.885 |
| HR(bpm) | 0.06 | [-0.14,0.34] | 0.412 | -0.13 | [-0.38,-0.02] | 0.034 |
| TG (mmol/L) | -0.06 | [-1.23,0.52] | 0.427 | -0.06 | [-1.34,0.46] | 0.334 |

BMI, body mass index; HbA1c, haemoglobin A1 c; HDL-C, high-density lipoprotein cholesterol; HR, heart rate; LDL-C, low-density lipoprotein cholesterol; SBP, systolic blood pressure; TG, total triglyceride.

**Supplementary Table S2** Multivariable Linear Regression Analysis of Average E/e' by Vitamin D Status.

|  | vitamin D deficiency | | | without vitamin D deficiency | | |
| --- | --- | --- | --- | --- | --- | --- |
| Predictor | β | 95%CI | P | β | 95%CI | P |
| NFS | 0.18 | [0.07,1.28] | 0.029 | 0.03 | [-0.62,0.92] | 0.705 |
| Sex | -0.19 | [-3.47,-0.63] | 0.005 | -0.16 | [-3.45,-0.36] | 0.016 |
| Age(years) | 0.20 | [0.01,0.13] | 0.032 | 0.03 | [-0.07,0.09] | 0.750 |
| SBP(mmHg) | 0.28 | [0.04,0.11] | ＜0.001 | 0.14 | [0.01,,0.08] | 0.027 |
| BMI(kg/m2) | 0.03 | [-0.13,0.21] | 0.636 | 0.01 | [-0.20,0.23] | 0.893 |
| Diabetes duration (years) | 0.30 | [0.11,0.29] | ＜0.001 | 0.08 | [-0.04,0.17] | 0.222 |
| HbA1C (%) | 0.12 | [-0.04,0.52] | 0.092 | 0.05 | [-0.21,0.49] | 0.433 |
| LDL-c (mmol/L) | -0.02 | [-0.75,0.55] | 0.765 | -0.02 | [-0.85,0.65] | 0.792 |
| HDL-c(mmol/L) | -0.01 | [-2.54,2.25] | 0.904 | -0.04 | [-3.35,1.79] | 0.549 |
| HR(bpm) | 0.15 | [0.00,0.11] | 0.035 | -0.03 | [-0.07,0.05] | 0.678 |
| TG (mmol/L) | -0.05 | [-0.25,0.13] | 0.525 | -0.05 | [-0.37,0.19] | 0.506 |

BMI, body mass index; HbA1c, haemoglobin A1 c; HDL-C, high-density lipoprotein cholesterol; HR, heart rate; LDL-C, low-density lipoprotein cholesterol; SBP, systolic blood pressure; TG, total triglyceride.
